# Gamified Cognitive Bias Interventions for Psychiatric Disorders: Protocol of a Systematic Review

**DOI:** 10.2196/10154

**Published:** 2018-10-16

**Authors:** Melvyn Zhang, Jiangbo Ying, Roger CM Ho

**Affiliations:** 1 National Addictions Management Service Institute of Mental Health Singapore Singapore; 2 Department of Psychological Medicine Yong Loo Lin School of Medicine National University of Singapore Singapore Singapore

**Keywords:** attention bias, cognitive bias, gamification, eHealth, mHealth

## Abstract

**Background:**

Cognitive bias modification has been increasingly studied in the past decade with reviews reporting the effectiveness of bias modification. Advances in electronic health and mobile health technologies have transformed how conventional cognitive bias modification is delivered. To date, gamification technologies and serious games have been widely evaluated in health care, and prior studies have reported the use of gamification for cognitive bias modification. However, no prior research, to date, has systematically evaluated the literature for gamified cognitive bias modification interventions.

**Objective:**

The proposed systematic review aims to review how gamification has been applied to cognitive bias modification interventions.

**Methods:**

A systematic review will be conducted. A search will be conducted on the respective databases till 2018. Selection of the studies will be determined by the Preferred Reporting Items for Systematic Review and Meta-Analysis guidelines. Quality assessment of the included studies will be assessed using the Cochrane Risk of Bias Tool. In addition, a narrative synthesis will be conducted.

**Results:**

We expect that the review will be completed 12 months from the publication of this protocol.

**Conclusions:**

The findings that arise from this review will be crucial as they will inform future research that seeks to integrate gamification technologies into existing conventional bias modification interventions.

**Registered Report Identifier:**

RR1-10.2196/10154

## Introduction

### Background

Gamification refers to the usage of game-designs features in nongame contexts [1]. In comparison, serious games refer to games that are explicitly created for nonrecreational purposes and are designed specifically for education, training, or behavioral modification [2]. Gamification, thus, differs from serious games, in that the main objective of gamification is in the creation of a game-like experience through the incorporation of various gaming mechanics and design. Some of the common gaming techniques include that of digital rewards, avatar, competition, social pressure, feedback, levels, achievement ranks, leaderboards, and three-dimensional environments [1]. Essentially, the main objective of introducing gamification elements is aimed at making interventions more engaging, enjoyable, and motivating for participants. Incorporating gaming elements changes the interaction between the individual and the app and might result in increased self-empowerment. Based on prior studies, it is known that gamification not only helps individuals to better engage with the intervention but also helps to increase individuals’ intrinsic and extrinsic motivation for intervention [3]. In addition, in some instances, gamification helps to improve skill sets and provides individuals with social support [3].

To date, gamification technologies and serious games have been widely evaluated in health care. Sardi et al [3] undertook a prior systematic review of gamification in electronic health and synthesized the evidence from 46 prior studies. Their review highlights that most of the current interventions are in the domains of chronic disease rehabilitation, physical activity, and mental health [3]. In addition, gamification, in the current context, serves mainly to be an extrinsic motivator for individuals [3]. Lau et al [2] in their recent meta-analytical study reviewed the evidence for serious games for psychiatric disorders. Based on their identified studies (10 studies, with studies involving the delivery of serious games through a computer), it was reported that serious games helped in the improvement of various psychiatric symptoms, with an effect size of 0.55, compared with controls [2]. While Lau et al’s prior review was limited to that of serious games and not specifically gamification approaches, it helps to demonstrate the potential effectiveness if gaming elements are included. Lumsden et al’s [4] review synthesized the current evidence for gamification, specifically for cognitive assessment and cognitive training. The authors, in their review, identified a total of 33 studies involving >31 gamified tasks and reported that researchers have considered gamification mainly to improve short- and long-term engagements and to make the task more attractive and potentially increasing the effect of cognitive training. Lumsden et al [4] recommended for there to be further validation of gamified tasks against a standard cognitive task and for studies to have the appropriate sample size, to better distinguish the effects of gamification on task performance. Of included studies in Lumsden et al’s [4] prior review, one focused on a specific form of cognitive retraining—attention bias modification. We will provide an overview of cognitive bias modification, the evidence for cognitive bias modification, the use of gamification technologies for cognitive bias modification, and define the rationale for this review.

Cognitive bias modification has been increasingly studied in the past decade. Cognitive biases include that of attention, approach or avoidance, and interpretative biases. Attentional biases refer to the preferential allocation of attention toward stimuli that are high in salience [5,6]. Closely related to attention biases are that of approach biases, which refer to automatic tendencies to reach out and approach stimuli with high salience [7]. In contrast, interpretative biases tend to result in individuals making negative evaluations of an ambiguous situation [8]. These biases have been posited to be involved in the psychopathologies of several psychiatric disorders, including anxiety disorders, alcohol use, and tobacco use disorders [9-13]. The presence of these biases implies that they could be subjected to manipulation and modification. Commonly, tasks such as that of the visual probe have been used for the modification of attention biases, and this involves the pairing of probes with the neutral stimulus (words or images) 100% of the time to retrain biases away from substance cues [14]. Tasks like the approach or avoidance are used for bias modification, by presenting cues in the push-away format and the neutral cues in a pull-closer format [15]. For modification of interpretations, this involves presenting individuals with ambiguous scenarios and with word fragments that help to disambiguate the scenarios positively [16]. There have been reviews conducted to date (Cristea et al [8] and Jones et al [17]) that have provided evidence pertaining to the overall effectiveness of bias modification. Cristea et al [8] identified trials including participants with alcohol or tobacco use disorders (25 trials) [18] in their review and reported that bias modification for both attentional and approach biases was moderately effective, with an effect size of 0.60 (Hedge 0.60) [19]. Despite there being changes in cognitive biases, the authors reported that there was no overall change in other symptomatology, such as that of cravings. The findings that Cristea et al arrived at might have been limited by the fact that they synthesized both clinical and nonclinical trials together, as highlighted by a commentary published in response to their meta-analysis. Jones et al [17] in their meta-meta-analyses found that cognitive bias modification was most effective or anxiety disorders, with the effect size for anxiety disorders ranging from 0.13 to 0.74 [17]. In addition, they reported the effect sizes of cognitive bias modification for depressive disorders to range between 0.35 and 0.85 and for appetitive disorders (defined to include eating disorders and addictive disorders) to range from 0.003 to 0.36 [17].

One of the main limitations of cognitive bias modification has been that the intervention has conventionally been confined to that of a laboratory, but that has changed in recent years. The advances in internet and mobile technologies led to these technologies being used for the delivery of cognitive bias modification. Wiers et al [20] administered the attention control training and approach bias retraining intervention using the internet among 136 problem drinkers and found a reduction in drinking across all intervention groups, even in the control group. In another study, William et al [21] delivered a Web-based cognitive bias modification training targeting imagery and interpretation bias among depressive individuals and found that the Web-based combined intervention (cognitive bias modification for interpretations together with internet cognitive behavioral therapy) was effective in reducing depression symptoms and distress symptoms, with an effect size (Cohen *d*) of 0.62-2.40. In particular, 27 participants had a clinically significant reduction in their symptoms following bias modification. These pioneering studies highlight the potential of bias modification delivered using the internet. The rapid advances in mobile health technologies have led to a further transformation of attention bias modification programs, as such mobile technologies are also being harnessed in the delivery of bias modification interventions. Clarke et al [22] reported that a mobile attention bias modification task was useful in helping to reduce the sleep-related threat, cognitive arousal, and help to improve insomnia symptoms; this was supported by electrophysiological measures that demonstrated that those who underwent the bias modification had better sleep quality. Apart from changes in the delivery mechanism for cognitive bias modification, a growing interest in gamification technologies has led to such technologies augmenting cognitive bias modification. For example, Dennis and O’Toole [18] reported how a single session of a gamified attention bias modification task was effective in reducing subjective anxiety and stress reactivity.

Boendermaker et al [23] in their prior review explored how gamification could help to address some of the issues of conventional bias modification tasks, particularly that of the motivation to train, given how repetitive bias modification interventions are; in addition, they highlighted several gamification approaches specifically for cognitive bias modification interventions and explored how published works have utilized some of these approaches. While Boendermaker et al’s [23] review provides a timely insight into how gamification strategies have been adapted for bias modification interventions, their review was not a systematic review, and no databases search was performed. There remains, to date, no prior research that has systematically evaluated the literature for gamified cognitive bias modification interventions. There is a need for evidence synthesis of these studies, for there to be an understanding of the effectiveness of a gamified approach for bias modification. Hence, this review is timely. Findings that arise from this review will be crucial as they will inform future research that seeks to integrate gamification technologies into existing conventional bias modification interventions.

### Research Aims

The proposed systematic review aims to review how gamification has been applied to cognitive bias modification interventions. We hope to address the following questions through this review: (1) What domains has gamification been applied in for cognitive bias modification interventions; (2) What is the effectiveness of gamification as applied to cognitive bias modification interventions? This will be assessed for by means of determining whether (1) there has been any motivational improvement over nongamified interventions; (2) there are changes in the bias scores; and (3) there is improvement in other secondary outcomes (eg, improvements in terms of anxiety or depression scores or a reduction in the total amount of alcohol consumed).

A systematic review will be undertaken to achieve the objective of this review. Studies identified will be reviewed by independent assessors and screened against our predefined inclusion and exclusion criteria. The Cochrane Risk of Bias Tool will be used for the assessment of the risk of biases in randomized trials that have been identified. Furthermore, the evidence will be synthesized using qualitative synthesis.

## Methods

### Search Strategy

To identify the relevant papers, the following search terminologies will be used: (“cognitive bias” OR “attention bias” OR “interpret* bais” OR “approach bias” OR “avoidance bias”) AND (“training” OR “modification” OR “practice” OR “therapy”) AND (“gamification” OR “game elements” OR “game” OR “gaming” or “Game mechanics”). The following databases will be searched: PubMed, Medical Literature Analysis and Retrieval System Online, PsycINFO, Science Direct, Scopus, EMBASE, and Cochrane CENTRAL. Databases will be searched from the year 2000 onwards to current, as prior to the year 2000, there were limited computer-based interventions. The search strategy will be modified for different databases. If full-text access is not available, the original authors will be contacted for their papers.

### Inclusion and Exclusion Criteria

Only English language papers will be included in this review. The inclusion criteria are as follows: (1) papers must describe a cognitive bias modification intervention; (2) the intervention utilized needs to be a novel gamified-like task; (3) papers included must be randomized studies; (4) participants must have underlying psychopathological symptoms; and (5) there needs to be a control or comparison group for comparison.

Papers will be excluded if they fulfill the following exclusion criteria: (1) papers are reviews, opinion pieces, or design documentations; (2) the intervention utilized is that of an existing over the shelf intervention; and (3) papers are nonrandomized studies.

### Condition or Domain Being Studied

This review will focus on all psychiatric disorders.

### Participants

Participants could be individuals recruited from either the community of from a treatment facility. Participants could be adolescents or adult participants. Participants need to have existing psychopathological symptoms (such as that of affective conditions). Participants do not need to be formally diagnosed with a psychiatric condition.

### Intervention

The intervention in this case is that of a cognitive bias intervention task, which could be that of the visual or dot-probe task and cognitive bias modification for interpretations.

### Comparisons

Participants will be compared with other participants who have received either treatment as usual or placebo or sham training.

### Outcome

For the outcome, the main (primary) outcome would be whether the gamified intervention has been effective, and if effective, the effect size for the intervention. For the secondary outcome, we will report if there is a reduction in the symptoms of specified psychiatric disorders and motivation to train or use the app.

### Data Extraction, Sorting, and Selection

All papers will be screened on the basis of their titles and abstract by two independent authors. Full copies of the shortlisted papers will then be evaluated against the inclusion and exclusion criteria. Any disagreement between the two authors will be resolved using discussion with the third author. An electronic document will be utilized to record systematically the reasons for inclusion and exclusion of each of the paper. This review will adhere to the reporting guidelines of the Preferred Reporting Items for Systematic Reviews and Meta-Analysis Protocols.

The following data will be systematically extracted from each paper and recorded on a standardized electronic data collation form and cross-checked by another independent assessor:

1. Publication details: authors(s) and study year
2. Study design and methods: study design, sample size, type of sample (treatment seeking or community cohorts), country in which study was conducted, demographics of the sample, diagnosis of participants, and methods using which diagnosis is made
3. Method of attention bias assessment and modification
4. Primary outcome: effectiveness of gamified attention bias modification
5. Secondary outcome: severity of underlying psychiatric condition and motivation to train or use the app

### Quality Assessment

The risk of bias assessment will be assessed by means of the Cochrane Collaboration Risk of Bias tool for randomized trials [24].

### Strategy for Data Integration or Synthesis

For this review, we will perform a narrative synthesis of the evidence. We will provide a summary of the domains or conditions in which gamification has been applied for cognitive bias modification. We will summarize the number of studies reporting the effectiveness of cognitive bias modification interventions. We will summarize and synthesize the evidence pertaining to the effectiveness. In addition, we will summarize the findings from all studies pertaining to whether there was a motivational improvement and whether there has been an improvement in other secondary outcomes reported.

## Results

We expect that the review will be completed 12 months from the publication of this protocol. We will report the results based on the identified outcomes as specified above.

## Discussion

### Principal Findings

To the best of our knowledge, this is the first planned study that will review the status of gamified cognitive bias modification interventions for psychiatric disorders. This planned review addresses the limitations of prior reviews, given that none of the prior studies has systematically evaluated the literature for gamified intervention. There is a need for evidence synthesis, as the evidence synthesis will help inform us about the types of psychiatric disorders that such features have been applied to. In addition, this review will help us in determining whether the gamified interventions have their basis on conventional cognitive bias modification tasks, such as that of the Stroop, visual probe, or cognitive bias modification for interventions. More importantly, the review will help us in the identification of common gaming elements that have been incorporated in published gamified interventions. This is of importance as it will help guide further research that would seek to develop a new gamified intervention for other forms of psychiatric disorders. If gamification is found to be effective in only some studies, this implies that there needs to be careful consideration of the appropriate gamification strategies to adopt.

### Conclusions

The findings that arise from this review will be crucial as they will inform future research that seeks to integrate gamification technologies into existing conventional bias modification interventions.

## References

[1] Hoffmann A, Christmann C, Bleser G (2017). Gamification in Stress Management Apps: A Critical App Review. JMIR Serious Games.

[2] Lau H, Smit J, Fleming T, Riper H (2016). Serious Games for Mental Health: Are They Accessible, Feasible, and Effective? A Systematic Review and Meta-analysis. Front Psychiatry.

[3] Sardi L, Idri A, Fernández-Alemán JL (2017). A systematic review of gamification in e-Health. J Biomed Inform.

[4] Lumsden J, Edwards E, Lawrence N, Coyle D, Munafò Marcus R (2016). Gamification of Cognitive Assessment and Cognitive Training: A Systematic Review of Applications and Efficacy. JMIR Serious Games.

[5] Field M, Cox W (2008). Attentional bias in addictive behaviors: a review of its development, causes, and consequences. Drug Alcohol Depend.

[6] Cox WM, Fadardi JS, Intriligator JM, Klinger E (2014). Attentional bias modification for addictive behaviors: clinical implications. CNS Spectr.

[7] Wiers R, Gladwin T, Hofmann W, Salemink E, Ridderinkhof K (2013). Cognitive Bias Modification and Cognitive Control Training in Addiction and Related Psychopathology. Clinical Psychological Science.

[8] Cristea I, Kok R, Cuijpers P (2016). The Effectiveness of Cognitive Bias Modification Interventions for Substance Addictions: A Meta-Analysis. PLoS One.

[9] Christiansen P, Schoenmakers TM, Field M (2015). Less than meets the eye: reappraising the clinical relevance of attentional bias in addiction. Addict Behav.

[10] Boettcher J, Leek L, Matson L, Holmes E, Browning M, MacLeod C (2013). Internet-based attention bias modification for social anxiety: a randomised controlled comparison of training towards negative and training towards positive cues. PLoS One.

[11] Macleod C (2012). Cognitive bias modification procedures in the management of mental disorders. Curr Opin Psychiatry.

[12] Boendermaker W, Sanchez MS, Boffo M, Wiers R (2016). Attentional Bias Modification With Serious Game Elementsvaluating the Shots Game. JMIR Serious Games.

[13] Field M, Duka T, Tyler E, Schoenmakers T (2009). Attentional bias modification in tobacco smokers. Nicotine Tob Res.

[14] Christiansen P, Mansfield R, Duckworth J, Field M, Jones A (2015). Internal reliability of the alcohol-related visual probe task is increased by utilising personalised stimuli and eye-tracking. Drug Alcohol Depend.

[15] Cousijn J, Goudriaan A, Wiers R (2011). Reaching out towards cannabis: approach-bias in heavy cannabis users predicts changes in cannabis use. Addiction.

[16] de Voogd E, de Hullu E, Burnett HS, Blackwell S, Wiers R, Salemink E (2017). Imagine the bright side of life: A randomized controlled trial of two types of interpretation bias modification procedure targeting adolescent anxiety and depression. PLoS One.

[17] Jones E, Sharpe L (2017). Cognitive bias modification: A review of meta-analyses. J Affect Disord.

[18] Dennis T, O'Toole L (2014). Mental Health on the Go: Effects of a Gamified Attention Bias Modification Mobile Application in Trait Anxious Adults. Clin Psychol Sci.

[19] Dennis-Tiwary TA, Denefrio S, Gelber S (2017). Salutary effects of an attention bias modification mobile application on biobehavioral measures of stress and anxiety during pregnancy. Biol Psychol.

[20] Wiers RW, Houben K, Fadardi JS, van Beek P, Rhemtulla M, Cox WM (2015). Alcohol cognitive bias modification training for problem drinkers over the web. Addict Behav.

[21] Williams A, Blackwell S, Mackenzie A, Holmes E, Andrews G (2013). Combining imagination and reason in the treatment of depression: a randomized controlled trial of internet-based cognitive-bias modification and internet-CBT for depression. J Consult Clin Psychol.

[22] Clarke PJF, Bedford K, Notebaert L, Bucks RS, Rudaizky D, Milkins BC, MacLeod C (2016). Assessing the Therapeutic Potential of Targeted Attentional Bias Modification for Insomnia Using Smartphone Delivery. Psychother Psychosom.

[23] Boendermaker WJ, Prins PJM, Wiers RW (2015). Cognitive Bias Modification for adolescents with substance use problems--Can serious games help?. J Behav Ther Exp Psychiatry.

[24] Higgins J, Altman D, Gøtzsche Peter C, Jüni Peter, Moher D, Oxman A, Savovic Jelena, Schulz Kenneth F, Weeks Laura, Sterne Jonathan A C, Cochrane Bias Methods Group, Cochrane Statistical Methods Group (2011). The Cochrane Collaboration's tool for assessing risk of bias in randomised trials. BMJ.

